# Hyperuricemia and gout increased the risk of long-term mortality in patients with heart failure: insights from the National Health and Nutrition Examination Survey

**DOI:** 10.1186/s12967-023-04307-z

**Published:** 2023-07-12

**Authors:** Yingdong Han, Yu Cao, Xinxin Han, Hong Di, Yue Yin, Juan Wu, Yun Zhang, Xuejun Zeng

**Affiliations:** 1 Department of family medicine, Peking Union Medical College Hospital, Chinese Academy of Medical Sciences, State Key Laboratory of Complex Severe and Rare Diseases, No. 1 Shuaifuyuan, Beijing, 100730 China; 2 Division of General Internal Medicine, Department of medicine, Peking Union Medical College Hospital, Chinese Academy of Medical Sciences, State Key Laboratory of Complex Severe and Rare Diseases, No. 1 Shuaifuyuan, Beijing, 100730 China

**Keywords:** Gout, Heart failure, Mortality, NHANES, Uric acid

## Abstract

**Background:**

The prevalence of hyperuricemia, gout, and heart failure (HF) is on the rise, and these conditions often share similar risk factors. The present study aimed to evaluate the relationship among hyperuricemia, gout, HF, and all-cause mortality.

**Methods:**

The data on nonpregnant participants aged ≥ 20 years with or without hyperuricemia, gout, and HF from the National Health and Nutrition Examination Survey 2001–2018 and 2007–2018 were included in this study. The binary logistic regression, Kaplan–Meier curve, Cox proportional-hazards model, and restricted cubic spline analysis were employed to evaluate the relationship among hyperuricemia, gout, HF, and all-cause mortality.

**Results:**

Of 204,179,060 and 223,702,171 weighted eligible participants, 40,044,228 (19.6%) and 9,158,600 (4.1%) had hyperuricemia and gout, respectively. Older age, diabetes, stroke, and coronary artery disease were the risk factors for HF among patients with hyperuricemia and gout. The median survival time was 7.00 years and 6.25 years and the 5-year survival rate was 59.9% and 55.9% for patients with HF and hyperuricemia and those with HF and gout, respectively. Patients with hyperuricemia or gout were 2.46 and 2.35 times more likely to have HF and 1.37 and 1.45 times more likely to experience all-cause mortality compared with those who did not exhibit these conditions. The restricted cubic spline showed a nonlinear correlation between uric acid levels and HF and a J-shaped correlation between uric acid levels and all-cause mortality.

**Conclusions:**

Ambulatory patients with hyperuricemia or gout were more likely to have HF compared with those without hyperuricemia or gout. Patients with HF with hyperuricemia or gout were more likely to experience all-cause mortality in the long-term follow-up.

**Supplementary Information:**

The online version contains supplementary material available at 10.1186/s12967-023-04307-z.

## Introduction

Serum uric acid (SUA) is the ultimate product of purine metabolism, which is mainly synthesized in the liver and excreted via the renal tract and intestines [1, 2]. Humans are exposed to > 50 times higher SUA concentrations than other mammals due to the lack of urate oxidase. The onset of hyperuricemia (HUA) is associated with the over-synthesis and underexcretion of SUA. HUA is the precursor of gout, which is one of the most common inflammatory arthritis, triggered by the crystallization of uric acid within the joints. The prevalence and disease burden of both HUA and gout have increased globally due to changes in dietary patterns and improved living conditions [3]. For example, a large-scale study in the USA showed that the prevalence of HUA and gout was 3.9% and 20.2%, respectively, in 2015–2016 [4]. In Europe, the gout prevalence ranged from 1 to 4% during 2003–2014 [5]. As an essential antioxidant in plasma, uric acid reacts with different oxidants and helps maintain blood pressure stability and resist oxidative stress [6]. HUA has a protective effect on neurodegenerative diseases, such as Parkinson’s and Alzheimer’s diseases [7]. However, intracellular uric acid is associated with high inflammation and increased oxidative stress [6, 8]. Besides the involvement of joints, HUA is a well-established prognostic marker in healthy individuals and patients with metabolic comorbidities, including metabolic syndrome, coronary artery disease (CAD), diabetes, and hypertension [9, 10].

Heart failure (HF) is a chronic phase of cardiac functional impairment secondary to many etiologies and one of the most rapidly occurring cardiovascular diseases (CVD) owing to the aging population and with an estimated prevalence of > 37.7 million individuals globally [11, 12]. It is a leading cause of hospitalization and readmissions among elderly people, is associated with increased mortality, and represents a burden to the healthcare system. The total medical expenses for patients with HF are expected to increase from $20.9 billion in 2012 to $53.1 billion by 2030 [13]. The etiology of HF varies considerably, and > 66% of HF cases can be attributed to ischemic heart disease, chronic obstructive pulmonary disease, hypertensive heart disease, and rheumatic heart disease [13].

The dual epidemic of HUA or gout with HF reflects the increased burden of cardiometabolic comorbidities. Older age, obesity, smoking, dyslipidemia, and diabetes are the independent risk factors for HF [12, 14]. Previous studies suggested that HUA and gout were associated with an increased risk of HF [15, 16]. Increased oxidative stress, endothelial dysfunction, inflammation, and insulin resistance might partially explain the increased incidence of HF. Some studies explored the relationship of HUA or gout with mortality [16–18]. The results varied considerably among studies due to different study populations, durations of follow-up, and study quality. Previous studies had limitations, such as a small sample size, short follow-up time, and an unrepresentative population. Also, no large-scale study has yet explored the long-term influence of HUA and gout on the mortality of patients with HF simultaneously.

The present study was performed on a nationally representative cohort of US adults whose data were obtained from the National Health and Nutrition Examination Survey (NHANES) 2001–2018. We conducted binary logistic regression and restricted cubic spline analysis to explore the relationship among HUA, gout, and HF. The survival analysis was performed, and a Cox proportional-hazards model was established to explore the effects of HUA and gout on the long-term mortality of patients with HF. Thus, this study provided a scientific basis for managing patients with HF complicated with HUA or gout.

## Materials and methods

### Data source

NHANES, conducted by the Centers for Disease Control and Prevention of America, collects the nutritional and health information of a representative sample of non-institutionalized US civilians. This national survey is carried out every 2 years using a multistage, stratified, sampling design to select participants. Written informed consent was obtained from every participant before the interview, and all the data were de-identified by the National Center for Health Statistics before making them publicly available. The survey consisted of a detailed in-home interview, a physical examination, and laboratory tests conducted at a specially equipped mobile examination center [19]. The study involved adult participants aged ≥ 20 years from nine NHANES cycles during 2001–2018 for analyzing the relationship between HUA and HF. Due to data limitations, we only involved participants in six NHANES cycles during 2007–2018 for the analysis between gout and HF. The investigation conformed with the principles outlined in the Declaration of Helsinki. Our study used publicly available de-identified data. The Research Ethics Review Board of the National Center for Health Statistics reviewed and approved all data collection protocols.

### Study population

In the study, we included all participants aged ≥ 20 years (unweighted *n* = 50,201 and 34,770). The pregnant participants at the time of examination and those with missing data on uric acid, gout, and HF were excluded. Also, those with ineligible data on mortality follow-up were excluded. Finally, 43,555 and 34,268 eligible participants were recruited in this study.

### Study variables

HUA was defined as the SUA level ≥ 420 μmol/L (7 mg/dL) in men and ≥ 360 μmol/L (6 mg/dL) in women [20]. Gout was defined as participants who answered “Yes” to the question, “Doctor ever told you that you had gout?” HF was defined as participants who answered “Yes” to the question, “Have you ever been told by a physician that you had congestive heart failure?”.

Variables in our study included age (divided into four groups, 20–39, 40–59, 60–79, and ≥ 80 years), sex, race, education level (< 9th grade, 9–11th grade, high school graduate, college degree, and college graduate or above), waist circumference, total cholesterol, triglyceride, body mass index (BMI) (< 18.5, 18.5–24.9, 25.0–29.9, and ≥ 30.0 kg/m^2^), marital status (married/living with a partner, widowed/divorced/separated, and never married), insurance status (yes or no), citizenship status (citizen or not a citizen of the USA), and smoking status (having smoked at least 100 cigarettes in life or not). Comorbidities, such as hypertension, diabetes, cancer, stroke, and CAD, were also recorded. The follow-up time was defined as the time from the interview to the death date or the end of the follow-up date.

### Outcomes

The relationship among HUA, gout, and HF was explored, the characteristics of participants with or without HF among those with established HUA or gout were compared, and the factors predicting those with concomitant HF were evaluated. As the main focus of our analysis, we evaluated the all-cause and cardiovascular mortalities in patients with HUA or gout compared with those without HUA and gout over a prolonged follow-up period. The National Center for Health Statistics has linked data from various surveys with death certificate records from the National Death Index with follow-up through NHANES 1999–2018. The Linked Mortality Files have been updated with mortality follow-up data through December 31, 2019 [21].

### Statistical analysis

All statistical analyses were conducted using SPSS 23 and R 4.1.0. The new sample weight (the original 2-year sample weight divided by 9 for the analysis between HUA and HF and the original 2-year sample weight divided by 6 for the analysis between gout and HF) was measured according to the analytical guidelines of NHANES [22]. The continuous variables were described with weighted means (standard error), and the categorical variables were described with unweighted numbers (weighted proportions). The participants’ characteristics between different groups were compared using the independent-sample *t* test or Mann–Whitney *U* test for continuous variables and the chi-square test for categorical variables. Weighted binary logistic regression was performed to determine the relationship between HUA or gout and the presence of cardiovascular comorbidities and HF. Age, sex, race, and BMI were adjusted in this study. Weighted logistic regression analysis was also used to determine the factors predicting HF in participants with established HUA or gout. Cox proportional-hazards models were used to estimate the hazard ratios and 95% confidence intervals (CIs) for all-cause mortality and cardiovascular mortality, adjusting for age, sex, race, and BMI. Restricted cubic spline analysis with three knots of the SUA levels was used to characterize the dose–response relationship between SUA levels and prevalence of HF as well as all-cause mortality. The overall survival time was illustrated using a Kaplan–Meier curve and compared with a log-rank test for patients with HF. A two-sided* P* value < 0.05 indicated a statistically significant difference.

## Results

The clinical characteristics of the involved participants in this study are summarized in Table 1 and Additional file 1: Table S1. A total of 43,555 (NHANES 2001–2018) and 34,268 (NHANES 2007–2018) unweighted participants met the eligibility criteria corresponding to 204,179,060 and 223,702,171 weighted, nationally representative participants, of whom 40,044,228 (19.6%) and 9,158,600 (4.1%) had HUA and gout. Participants with HUA or gout were older [51.04 (0.28) vs 46.48 (0.20) and 61.55 (0.48) vs 47.08 (0.23)], predominantly men (57.1% vs 46.9% and 67.8% vs 47.8%), likely to be obese (54.7% vs 30.7% and 55.0% vs 36.4%), smokers (49.0% vs 45.5% and 56.5% vs 43.7%), and covered by health insurance (84.2% vs 81.7% and 91.8% vs 82.0%). Patients with HUA or gout were likely to be complicated with cardiometabolic comorbidities, diabetes (13.5% vs 8.1% and 26.5% vs 9.2%), hypertension (48.9% vs 26.8% and 68.4% vs 30.7%), CAD (5.5% vs 3.0% and 12.9% vs 3.0%), HF (5.7% vs 1.6% and 10.5% vs 2.1%), and stroke (4.6% vs 2.4% and 8.3% vs 2.7%). The multivariable binary logistic regression analysis (Table 2 and Additional file 1: Table S2) showed that patients with HUA or gout were likely to have HF [2.46 (2.10–2.89) and 2.35 (1.83–3.02)], CAD [1.33 (1.13–1.56) and 1.85 (1.49–2.30)], and stroke [1.43 (1.23–1.65) and 1.63 (1.23–2.17)], adjusting for age, sex, race, and BMI.

**Table 1:** Baseline and demographic characteristics of participants with and without hyperuricemia in NHANES, 2001–2018

Variables	Normal uric acid (unweighted n = 34,556)	Hyperuricemia (unweighted n = 8999)	*P* value
Weighted, n (%)	164,134,832 (80.4)	40,044,228 (19.6)	
Age (mean [SE])^a^	46.48 (0.20)	51.04 (0.28)	< 0.001
Age groups (%)			< 0.001
20–39	11,949 (37.8)	2214 (30)	
40–59	11,713 (39.1)	2660 (35.2)	
60–79	8807 (19.5)	3159 (27.5)	
≥ 80	2087 (3.6)	966 (7.2)	
Gender (%)			< 0.001
Men	16,616 (46.9)	4987 (57.1)	
Women	17,940 (53.1)	4012 (42.9)	
Race (%)			< 0.001
Mexican American	6209 (5.9)	1050 (8.8)	
Other Hispanic	3109 (4.2)	585 (5.7)	
Non-Hispanic White	15,231 (70.3)	4243 (68.2)	
Non-Hispanic Black	6655 (12.4)	2244 (10.2)	
Other race	3352 (7.2)	877 (7.0)	
Education level (%)			< 0.001
Less than 9th grade	4044 (5.8)	997 (5.9)	
9–11th grade	4927 (10.7)	1311 (11.0)	
High school graduate	7874 (23.3)	2232 (26.1)	
College or AA degree	9853 (30.8)	2701 (33.1)	
College graduate or above	7817 (29.4)	1746 (23.8)	
Citizenship status (%)			< 0.001
Citizen by birth or naturalization	29,196 (90.3)	8192 (93.9)	
Not a citizen of the US	5291 (9.6)	797 (6.0)	
Marital status (%)			
Married/living with partner	21,000 (64.5)	5111 (60.9)	< 0.001
Widowed/divorced/separated	7347 (17.7)	2471 (22.3)	
Never married	6190 (17.8)	1413 (16.7)	
Insurance status (%)			< 0.001
Yes	26,830 (81.7)	7464 (84.2)	
No	7621 (18.0)	1507 (15.5)	
BMI (%)			< 0.001
< 18.5	631 (1.9)	44 (0.4)	
18.5–24.9	10,690 (32.6)	1199 (12.6)	
25–29.9	11,742 (33.6)	2721 (30.4)	
≥ 30.0	10,948 (30.7)	4818 (54.7)	
Waist circumference^a^	96.45 (0.18)	107.66 (0.31)	< 0.001
Smoking (%)	15,546 (45.5)	4455 (49.0)	< 0.001
Total cholesterol	5.03 (0.011)	5.20 (0.017)	< 0.001
Triglycerides	1.61 (0.013)	2.12 (0.026)	< 0.001
Hypertension (%)	10,572 (26.8)	4910 (48.9)	< 0.001
Diabetes (%)	3883 (8.1)	1569 (13.5)	< 0.001
Cancer (%)	3015 (9.1)	1085 (11.9)	< 0.001
Stroke (%)	1127 (2.4)	561 (4.6)	< 0.001
Heart failure (%)	790 (1.6)	681 (5.7)	< 0.001
Coronary artery disease (%)	1257 (3.0)	596 (5.5)	< 0.001
Heart disease (combination of CAD, stroke, heart failure) (%)	2550 (5.8)	1401 (12.2)	< 0.001

*CAD* coronary artery disease

^a^Data are presented as unweighted n (weighted percentage) for categorical variables and weighted means and standard errors for continuous variables

**Table 2:** Odds ratios (95% CIs) for the prevalence of common comorbidities in participants with hyperuricemia compared with participants without hyperuricemia

Variables	Unadjusted OR	95% CIs		*P* value	Adjusted OR	95% CIs		*P* value
HF	3.67	3.15	4.27	< 0.001	2.46	2.10	2.89	< 0.001
CAD	1.89	1.63	2.20	< 0.001	1.33	1.13	1.56	0.001
Stroke	1.98	1.75	2.25	< 0.001	1.43	1.23	1.65	< 0.001
Heart disease	2.27	2.04	2.51	< 0.001	1.59	1.42	1.78	< 0.001
Diabetes	1.76	1.61	1.93	< 0.001	1.12	1.01	1.23	0.030
Hypertension	2.62	2.47	2.78	< 0.001	1.92	1.79	2.07	< 0.001
Obesity	2.80	2.62	2.99	< 0.001	2.98	2.78	3.19	< 0.001
Cancer	1.34	1.21	1.47	< 0.001	1.05	0.95	1.17	0.322

Age, gender, race and BMI were adjusted

*CIs* confidence intervals *HF* heart failure *CAD* coronary artery disease

Among patients with HUA or gout, those complicated with HF were older [67.97 (0.63) vs 50.02 (0.28) and 67.79 (0.99) vs 60.82 (0.52)], with a high waist circumference [112.65 (0.93) vs 107.38 (0.30) and 112.23 (1.61) vs 109.46 (0.75)], likely to be obese (62.0% vs 55.4% and 65.1% vs 55.3%), smokers (61.3% vs 48.2% and 64.0% vs 55.6%), and covered by health insurance (84.2% vs 81.7% and 91.8% vs 82.0%), and had a high prevalence of cardiometabolic comorbidities, including obesity (62.0% vs 55.4% and 65.1% vs 55.3%), diabetes (40.4% vs 11.9% and 52.9% vs 23.4%), hypertension (81.1% vs 47.1% and 79.9% vs 67.1%), stroke (19.8% vs 3.7% and 24.5% vs 6.3%), and CAD (35.9% vs 3.7% and 44.8% vs 9.1%) (Table 3 and Additional file 1: Table S3). In multivariable logistic regression models, patients with HUA or gout complicated with HF were likely to be older [OR (95% CIs): 1.05 (1.04–1.06) and 1.03 (1.00–1.05)], complicated with diabetes [OR (95% CIs): 2.09 (1.63–2.68) and 2.47 (1.67–3.65)], CAD [OR (95% CIs): 7.03 (5.18–9.54) and 6.45 (3.94–10.53)], and stroke [OR (95% CIs): 2.20 (1.48–3.29) and 3.22 (1.87–5.53)] (Table 4 and Additional file 1: Table S4).

**Table 3:** Baseline and demographic characteristics of participants with and without heart failure among those with hyperuricemia

Variables	No HF (Unweighted n = 8333)	HF (Unweighted n = 681)	*P* value
Weighted, n (%)	37,813,728 (94.3)	2,283,237 (5.7)	
Age (mean [SE])^a^	50.02 (0.28)	67.97 (0.63)	< 0.001
Age groups (%)			< 0.001
20–39	2206 (31.7)	11 (2.1)	
40–59	2545 (36.1)	120 (21.2)	
60–79	2797 (26.0)	369 (52.8)	
≥ 80	785 (6.2)	182 (23.9)	
Gender (%)			< 0.001
Men	4632 (57.7)	363 (48.3)	
Women	3701 (42.3)	318 (51.7)	
Race (%)			< 0.001
Mexican American	1002 (6.1)	50 (2.9)	
Other Hispanic	546 (4.3)	40 (3.2)	
Non-Hispanic White	3866 (70.0)	379 (74.6)	
Non-Hispanic Black	2062 (12.2)	185 (15.5)	
Other race	857 (7.4)	27 (3.9)	
Education level (%)			< 0.001
Less than 9th grade	893 (5.6)	108 (11.4)	
9–11th grade	1186 (10.7)	126 (16.0)	
High school graduate	2053 (25.9)	181 (28.7)	
College or AA degree	2511 (33.2)	192 (31.0)	
College graduate or above	1678 (24.5)	73 (12.8)	
Citizenship status (%)			< 0.001
Citizen by birth or naturalization	7544 (93.6)	661 (98.5)	
Not a citizen of the US	779 (6.3)	20 (1.5)	
Marital status (%)			
Married/living with partner	4793 (61.4)	330 (53.8)	< 0.001
Widowed/divorced/separated	2165 (21.2)	307 (40.7)	
Never married	1370 (17.4)	44 (5.5)	
Insurance status (%)			< 0.001
Yes	6388 (83.8)	639 (93.9)	
No	1468 (16.1)	40 (6.0)	
BMI (%)			< 0.001
< 18.5	42 (0.4)	2 (0.3)	
18.5–24.9	1115 (12.9)	85 (11.7)	
25–29.9	2552 (31.3)	177 (26.0)	
≥ 30.0	4445 (55.4)	379 (62.0)	
Waist circumference ^a^	107.38 (0.30)	112.65 (0.93)	< 0.001
Smoking (%)	4048 (48.2)	441 (61.3)	< 0.001
Total cholesterol	5.24 (0.018)	4.59 (0.058)	< 0.001
Triglycerides^a^	2.13 (0.027)	1.92 (0.059)	< 0.001
Uric acid^a^	438.94 (0.76)	468.24 (3.77)	< 0.001
Hypertension (%)	4345 (47.1)	572 (81.1)	< 0.001
Diabetes (%)	1280 (11.9)	292 (40.4)	< 0.001
Cancer (%)	934 (10.9)	151 (27.8)	< 0.001
Stroke (%)	424 (3.7)	137 (19.8)	< 0.001
Coronary artery disease (%)	347 (3.7)	250 (35.9)	< 0.001

^a^Data are presented as unweighted n (weighted percentage) for categorical variables and weighted means and standard errors for continuous variables

**Table 4:** Odds ratios (95% CIs) for risk factors for concomitant HF in participants with hyperuricemia

Variables	Adjust OR (95%CIs)	*P* value
Age (years)	1.05 (1.04–1.06)	< 0.001
Female	0.82 (0.62–1.07)	0.140
Mexican American (ref)		
Other Hispanic	1.44 (0.76–2.76)	0.262
Non-Hispanic White	1.13 (0.76–1.66)	0.547
Non-Hispanic Black	1.55 (0.99–2.44)	0.056
Other race	1.04 (0.52–2.07)	0.917
No insurance	1.05 (0.64–1.73)	0.845
Less than 9th grade (ref)		
9–11th grade	0.96 (0.64–1.43)	0.85
High school graduate	0.84 (0.59–1.20)	0.330
College or AA degree	0.81 (0.54–1.23)	0.324
College graduate or above	0.40 (0.24–0.67)	< 0.001
Not a citizen of the US	0.35 (0.19–0.63)	< 0.001
Married/Living with partner (ref)		
Widowed/divorced/separated	1.20 (0.94–1.53)	0.152
Never married	1.07 (0.63–1.84)	0.791
Hypertension	0.75 (0.52–1.07)	0.113
Diabetes	2.09 (1.63–2.68)	< 0.001
Cancer	1.58 (1.12–2.22)	0.009
Stroke	2.20 (1.48–3.29)	< 0.001
Coronary artery disease	7.03 (5.18–9.54)	< 0.001
Obesity	1.48 (1.16–1.90)	0.002

*CIs* confidence intervals *HF* heart failure

The results of the Cox proportional-hazards models are presented in Tables 5, 6 and Additional file 1: Tables S5, S6. During a median follow-up of 9.67 (5.42–13.92) and 7.17 (4.08–10.08) years, the adjusted risk of all-cause mortality and cardiovascular mortality was higher in patients with HUA and gout compared with those without HUA or gout [all-cause mortality: OR (95% CIs): 1.43 (1.35–1.52) and 1.47 (1.31–1.65); cardiovascular mortality: OR (95% CIs): 1.61 (1.44–1.79) and 1.53 (1.23–1.90)]. Similarly, the adjusted risk of all-cause mortality and cardiovascular mortality was higher in patients with HF and HUA and those with HF and gout compared with those without HUA, gout, and HF [all-cause mortality: OR (95% CIs): 3.49 (3.12–3.90) and 3.59 (2.93–4.38); cardiovascular mortality: OR (95% CIs): 5.42 (4.50–6.52) and 5.11 (3.63–7.18)]. Patients with HF and HUA and those with HF and gout had a higher risk of all-cause mortality compared with patients with HF but without HUA or gout [OR (95% CIs): 1.37 (1.18–1.60) and 1.45 (1.15–1.82), respectively]. Finally, the adjusted risk of all-cause mortality was higher in patients with HF and gout accompanied by HUA [OR (95% CIs): 1.78 (1.32–2.40)], whereas it became insignificant in patients with HF and gout without HUA [OR (95% CIs): 1.17 (0.83–1.65)] compared with patients with HF without gout.

**Table 5:** HR (95% CIs) for all-cause mortality according to heart failure and hyperuricemia among participants in NHANES 2001–2018

Variables	Adjusted HR (95% CIs)	*P* value
No HUA (ref)		
HUA	1.43 (1.35–1.52)	< 0.001
No HUA or HF (ref)		
HUA without HF	1.36 (1.28–1.45)	< 0.001
HF without HUA	2.55 (2.28–2.85)	< 0.001
HUA with HF	3.49 (3.12–3.90)	< 0.001
HF without HUA (ref)		
HF with HUA	1.37 (1.18–1.60)	< 0.001

Age, gender, race and BMI were adjusted

*CIs* confidence intervals *HF* heart failure *HUA* hyperuricemia *HR* hazard ratio

**Table 6:** HR (95% CIs) for cardiovascular mortality according to heart failure and hyperuricemia among participants in NHANES 2001–2018

Variables	Adjusted HR (95% CIs)	*P* value
No HUA (ref)		
HUA	1.61 (1.44–1.79)	< 0.001
No HUA or HF (ref)		
HUA without HF	1.46 (1.29–1.66)	< 0.001
HF without HUA	3.82 (3.16–4.62)	< 0.001
HUA and HF	5.42 (4.50–6.52)	< 0.001

*CIs* confidence intervals *HF* heart failure *HUA*: hyperuricemia, H*R* hazard ratios

**Fig. 1:**
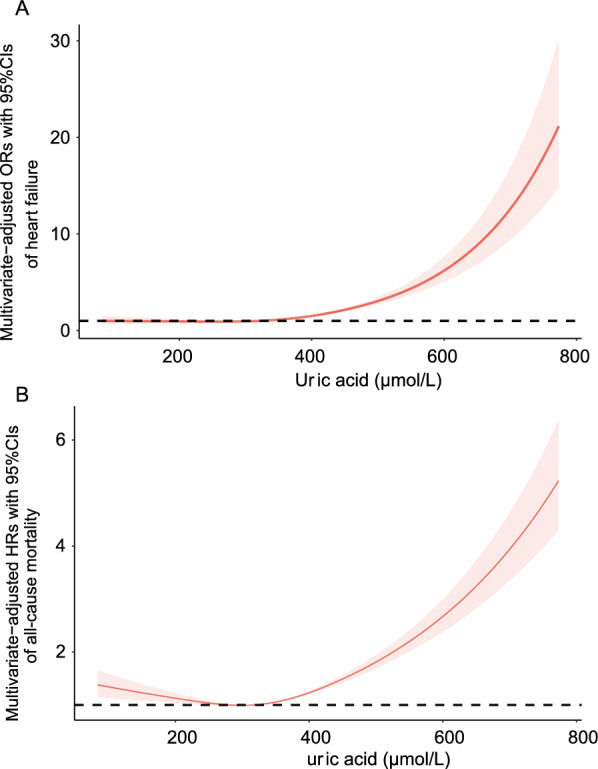
**A** Dose–response relationship between SUA level (μmol/L) and the risk of HF detected using the restricted cubic spline model. **B** Dose–response relationship between SUA level (μmol/L) and all-cause mortality detected using the restricted cubic spline model. The restricted cubic spline model was adjusted for age, sex, race, and BMI

The dose–response relationship between SUA and the risk of HF is presented in Fig. 1A. The restricted cubic spline suggested that the uric acid levels were positively associated with the risk of HF in US adults aged ≥ 20 years and showed a nonlinear correlation (*P* < 0.001, *P* for nonlinearity < 0.001). The dose–response relationship between SUA and the risk of all-cause mortality is presented in Fig. 1B. The restricted cubic spline revealed a J-shaped association between SUA and all-cause mortality (*P* < 0.001, *P* for nonlinearity < 0.001).

**Fig. 2:**
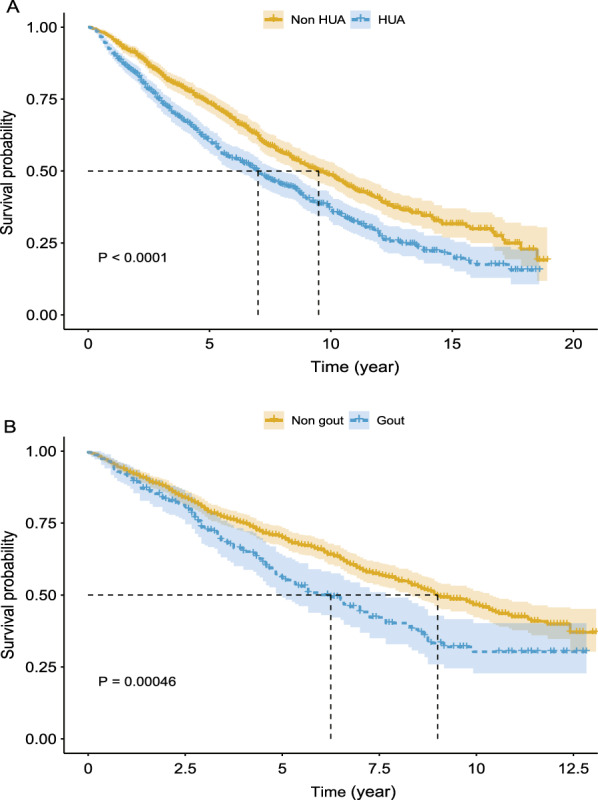
Kaplan–Meier survival curves for all-cause mortality in patients with HF complicated with HUA (**A**) and gout (**B**)

The Kaplan–Meier survival curves for unadjusted all-cause mortality of patients with HF are presented in Fig. 2A, B. The 5-year survival rate was 59.9% and 55.9% for patients in the HF + HUA and HF + gout groups, respectively. The median survival time was 7.00 years and 6.25 years for patients with HF with HUA or gout, respectively. The participants in the HF + HUA and HF + gout groups displayed significantly greater all-cause mortality than those in the HF without HUA or gout group (both log-rank *P* values < 0.001).

## Discussion

The present study found that ambulatory patients with HUA, gout, or HF had a high burden of cardiometabolic comorbidities, including obesity, hypertension, diabetes, CAD, and stroke. In multivariate binary logistic regression models, patients with HUA or gout were 2.46 and 2.35 times more likely to have HF compared with patients without HUA or gout, respectively. Among patients with HUA or gout, older age, diabetes, stroke, and CAD were the risk factors for HF. In contrast, a college graduate or above degree served as a protective factor for HF. In multivariate Cox proportional-hazard models and Kaplan–Meier survival curves, HF patients with HUA or gout had a higher risk of all-cause and cardiovascular mortalities after a median follow-up of 9.67 and 7.17 years. The restricted cubic spline showed a nonlinear correlation between SUA and the risk of HF and a J-shaped association between SUA and all-cause mortality.

In this study, we used a large, nationally representative, and long-term cohort of NHANES, which increased the statistical strength to provide a reliable result. The study held significant public health importance. Next, we expanded the current understanding of the possible relationship between HUA and gout with the prevalence of HF and clinical endpoints. Linking the NHANES data with mortality data from the National Center for Health Statistics provided the long-term outcomes of our study population. Except for the effects of HUA and gout on HF, we explored the risk factors associated with HF among patients with HUA and gout and the dose–response relationship between SUA and prevalence and all-cause mortality of HF. This study was novel in simultaneously investigating the effects of HUA and gout on the risk and long-term prognosis of HF.

Currently, the role of SUA in HF incidence and mortality is poorly understood. Hypoxia- and ischemia-induced upregulation of xanthine oxidase (XO) activity in a failing heart leads to increased purine degradation and oxidative stress [23, 24]. XO-derived reactive oxygen species (ROS) may account for a range of detrimental processes in the pathophysiology of HF. XO is also correlated with NADPH oxidase, another major ROS producer [16]. SUA exerts many deleterious effects on cells, and thus it may be directly involved in the pathophysiology of CVD. The depletion of nitric oxide and endothelial dysfunction, promotion of local inflammation and pro-oxidant activity, increased oxidative stress, and potentiation of vasoconstrictor and proliferative vascular stimuli are the most accepted pathophysiological mechanisms of SUA involvement in the development of CVD. SUA may serve as an internal danger signal that stimulates the innate immune response, which plays a role in HF and atherosclerosis [20]. Additionally, SUA is associated with multiple cardiovascular risk factors, such as insulin resistance, hypertension, metabolic syndrome, and chronic kidney disease [25–28].

In previous studies using the Framingham Offspring cohort and health check-up data [15, 29], Holme and Krishnan found that elevated SUA level was associated with an increased incidence of HF. A previous study conducted by Colantonio found that gout is associated with an increased risk of HF [16]. These results were consistent with the previous findings and showed that the increased risk of HF translated into poor outcomes. We also explored the risk factors associated with increased odds of HF among patients with HUA and gout, such as older age, diabetes, stroke, and CAD. Stamp et al. found that patients with gout were at an increased risk of readmission for HF but not all-cause mortality [18]. However, in the present study, we used a large-scale representative cohort of NHANES and found that patients with HF complicated with HUA or gout had a high risk of all-cause mortality after long-term follow-up, which was consistent with the findings of Pilote [30]. Further, we explored the effects of HUA and gout on cardiovascular mortality. These findings provided valuable epidemiological evidence for identifying the modifiable risk factors and contained the emerging dual epidemic of HUA, gout, and HF.

Nevertheless, the present study had some limitations. The SUA level was measured only once, whereas uric acid was not static but a dynamic variable, which might be affected by the diet, leading to some bias. It was impossible to include all potential confounding factors due to limited data availability. Since the data regarding left ventricular ejection fraction and HF phenotype were not reported in NHANES, the diagnosis of HF was determined using a questionnaire, which might have led to recall bias. Also, the analysis of cardiovascular mortality in patients with HF was carried out on a small sample to derive meaningful conclusions and was not reported.

## Conclusions

Ambulatory patients with HUA or gout were 2.46 and 2.35 times more likely to have HF and patients with HF and HUA or gout were 1.37 and 1.45 times more likely to experience all-cause mortality in the long-term follow-up compared with those without HUA or gout. Therefore, attention should be paid to the SUA levels and joint symptoms in patients with HF.

## Supplementary Information

**Additional file 1:** **Table S1.** Baseline and demographic characteristics of participants with and without gout in NHANES, 2007-2018. **Table S2**. Odds ratios (95% CIs) for the prevalence of cardiovascular comorbidities in participants with gout compared with participants without gout. **Table S3.** Baseline and Demographic characteristics of participants with and without heart failure among those with gout. **Table S4**. Odds ratios (95%CIs) for risk factors for concomitant HF in participants with gout. **Table S5.** HR (95% CIs) for all-cause mortality according to heart failure and gout among participants in NHANES 2007-18. **Table S6**. HR (95% CIs) for cardiovascular mortality according to heart failure and gout among participants in NHANES 2007-18.

## Data Availability

The datasets supporting the conclusions of this article was available in the public repository as described below. The authors do not own the data. National Health and Nutrition Examination Survey data are available from the National Center for Health Statistics (http://www.cdc.gov/nchs/nhanes/nhanes_questionnaires.htm).

## Abbreviations

BMI: Body mass index
CAD: Coronary artery disease
CIs: Confidence intervals
CVD: Cardiovascular disease
HF: Heart failure
HUA: Hyperuricemia
NHANES: National Health and Nutrition Examination Surveys
ROS: Reactive oxygen species
SUA: Serum uric acid
XO: Xanthine oxidase
