# Supplementary material for: Hyperuricemia and gout increased the risk of long-term mortality in patients with heart failure: insights from the National Health and Nutrition Examination Survey
Source: J Transl Med. 2023 Jul 12;21:463. doi: 10.1186/s12967-023-04307-z (PMC10339518; doi:10.1186/s12967-023-04307-z)
Supplement: Supplementary file 1 — Additional file 1: Table S1. Baseline and demographic characteristics of participants with and without gout in NHANES, 2007-2018. Table S2. Odds ratios (95% CIs) for the prevalence of cardiovascular comorbidities in participants with gout compared with participants without gout. Table S3. Baseline and Demographic characteristics of participants with and without heart failure among those with gout. Table S4. Odds ratios (95%CIs) for risk factors for concomitant HF in participants with gout. Table S5. HR (95% CIs) for all-cause mortality according to heart failure and gout among participants in NHANES 2007-18. Table S6. HR (95% CIs) for cardiovascular mortality according to heart failure and gout among participants in NHANES 2007-18. [file 12967_2023_4307_MOESM1_ESM.docx]

**Table S1.** Baseline and demographic characteristics of participants with and without gout in NHANES, 2007-2018.

| Variables | No gout  (Unweighted n= 32624) | Gout  (Unweighted n = 1644) | *P* value |
| --- | --- | --- | --- |
| Weighted n (%) | 214543571 (95.9) | 9158600 (4.1) |  |
| Age (mean [S.E]) * | 47.08 (0.23) | 61.55 (0.48) | <0.001 |
| Age groups (%) |  |  | <0.001 |
| 20-39 | 11089 (37.2) | 93 (7.1) |  |
| 40-59 | 10851 (37.6) | 423 (33.5) |  |
| 60-79 | 8593 (21.0) | 868 (47.5) |  |
| ≥80 | 2091 (4.2) | 260 (11.9) |  |
| Gender (%) |  |  | <0.001 |
| Men | 15650 (47.8) | 1130 (67.8) |  |
| Women | 16974 (52.2) | 514 (32.2) |  |
| Race (%) |  |  | <0.001 |
| Mexican American | 4990 (8.7) | 116 (3.6) |  |
| Other Hispanic | 3515 (6.0) | 93 (2.9) |  |
| Non-Hispanic White | 13113 (65.8) | 784 (72.5) |  |
| Non-Hispanic Black | 6910 (11.3) | 457 (13.2) |  |
| Other race | 4096 (8.2) | 194 (7.8) |  |
| Education level (%) |  |  | <0.001 |
| Less than 9th grade | 3499 (5.5) | 197 (6.2) |  |
| 9-11th grade | 4605 (10.5) | 254 (11.0) |  |
| High school graduate | 7422 (23.0) | 397 (24.5) |  |
| College or AA degree | 9524 (31.2) | 464 (31.4) |  |
| College graduate or above | 7528 (29.7) | 332 (26.9) |  |
| Citizenship status (%) |  |  | <0.001 |
| Citizen by birth or naturalization | 27668 (90.4) | 1558 (96.4) |  |
| Not a citizen of the US | 4861 (9.4) | 85 (3.5) |  |
| Marital status (%) |  |  | <0.001 |
| Married/Living with partner | 19031 (62.4) | 1016 (69.0) |  |
| Widowed/divorced/separated | 7338 (18.5) | 506 (24.3) |  |
| Never married | 6231 (19.1) | 121 (6.6) |  |
| Insurance Status (%) |  |  | <0.001 |
| Yes | 25484 (82.0) | 1498 (91.8) |  |
| No | 7094 (17.9) | 144 (8.1) |  |
| BMI (%) |  |  | <0.001 |
| <18.5 | 515 (1.6) | 12 (0.4) |  |
| 18.5-24.9 | 8595 (28.4) | 229 (12.8) |  |
| 25-29.9 | 10186 (32.6) | 474 (29.6) |  |
| ≥30.0 | 11619 (36.4) | 827 (55.0) |  |
| Waist circumference * | 98.83 (0.22) | 109.71 (0.74) | <0.001 |
| Smoking (%) | 14170 (43.7) | 949 (56.5) | <0.001 |
| Total cholesterol * | 5.01 (0.013) | 4.83 (0.047) | <0.001 |
| Triglycerides * | 1.71 (0.015) | 2.18 (0.064) | <0.001 |
| Uric acid * | () | () | <0.001 |
| Hypertension (%) | 11335 (30.7) | 1203 (68.4) | <0.001 |
| Diabetes (%) | 4037 (9.2) | 543 (26.5) | <0.001 |
| Cancer (%) | 3017 (9.9) | 327 (22.1) | <0.001 |
| Stroke (%) | 1197 (2.7) | 182 (8.3) | <0.001 |
| Heart failure (%) | 931 (2.1) | 240 (10.5) | <0.001 |
| Coronary artery disease (%) | 1195 (3.0) | 222 (12.9) | <0.001 |
| Heart disease (combination of CAD, stroke, heart failure) (%) | 2650 (6.4) | 474 (23.7) | <0.001 |

*Data are presented as unweighted n (weighted percentage) for categorical variables and weighted means and standard errors for continuous variables.

CAD: coronary artery disease.

**Table S2**. Odds ratios (95% CIs) for the prevalence of cardiovascular comorbidities in participants with gout compared with participants without gout.

| Variables | Unadjusted  OR | 95% CIs | | *P* value | Adjusted  OR | 95% CIs | | *P* value |
| --- | --- | --- | --- | --- | --- | --- | --- | --- |
| HF | 5.46 | 4.43 | 6.73 | <0.001 | 2.35 | 1.83 | 3.02 | <0.001 |
| CAD | 4.69 | 3.81 | 5.78 | <0.001 | 1.85 | 1.49 | 2.30 | <0.001 |
| Stroke | 3.22 | 2.54 | 4.09 | <0.001 | 1.63 | 1.23 | 2.17 | 0.001 |
| Heart disease | 4.58 | 3.93 | 5.34 | <0.001 | 2.05 | 1.73 | 2.42 | <0.001 |
| Diabetes | 3.53 | 3.05 | 4.09 | <0.001 | 1.70 | 1.45 | 1.98 | <0.001 |
| Hypertension | 4.89 | 4.18 | 5.74 | <0.001 | 2.44 | 2.02 | 2.93 | <0.001 |
| Obesity | 2.22 | 1.89 | 2.60 | <0.001 | 2.27 | 1.92 | 2.69 | <0.001 |
| Cancer | 2.58 | 2.14 | 3.10 | <0.001 | 1.47 | 1.21 | 1.78 | <0.001 |

CIs: confidence intervals; HF: heart failure; CAD: coronary artery disease.

Age, gender, race and BMI were adjusted.

**Table S3.** Baseline and Demographic characteristics of participants with and without heart failure among those with gout.

| Variables | No HF  (Unweighted n= 1404) | HF  (Unweighted n = 240) | *P* value |
| --- | --- | --- | --- |
| Weighted n (%) | 8195109 (89.5) | 963491 (10.5) |  |
| Age (mean [S.E]) * | 60.82 (0.52) | 67.79 (0.99) | <0.001 |
| Age groups (%) |  |  | <0.001 |
| 20-39 | 89 (7.7) | 4 (2.6) |  |
| 40-59 | 377 (34.9) | 46 (21.1) |  |
| 60-79 | 733 (46.9) | 135 (53.1) |  |
| ≥80 | 205 (10.5) | 55 (23.1) |  |
| Gender (%) |  |  | 0.767 |
| Men | 967 (68.6) | 163 (61.9) |  |
| Women | 437 (31.4) | 77 (38.1) |  |
| Race (%) |  |  | <0.001 |
| Mexican American | 106 (3.7) | 10 (2.2) |  |
| Other Hispanic | 77 (2.7) | 16 (5.0) |  |
| Non-Hispanic White | 669 (73.5) | 115 (64.2) |  |
| Non-Hispanic Black | 368 (12.0) | 89 (23.6) |  |
| Other race | 184 (8.1) | 10 (5.0) |  |
| Education level (%) |  |  | 0.001 |
| Less than 9th grade | 159 (5.7) | 38 (10.7) |  |
| 9-11th grade | 213 (10.4) | 41 (16.0) |  |
| High school graduate | 333 (23.6) | 64 (32.5) |  |
| College or AA degree | 391 (31.8) | 73 (28.4) |  |
| College graduate or above | 308 (28.6) | 24 (12.4) |  |
| Citizenship status (%) |  |  | 0.514 |
| Citizen by birth or naturalization | 1327 (96.2) | 231 (97.8) |  |
| Not a citizen of the US | 76 (3.6) | 9 (2.2) |  |
| Marital status (%) |  |  |  |
| Married/Living with partner | 891 (71.0) | 125 (52.9) | 0.001 |
| Widowed/divorced/separated | 407 (22.4) | 99 (40.3) |  |
| Never married | 105 (6.6) | 16 (6.8) |  |
| Insurance Status (%) |  |  | 0.013 |
| Yes | 1266 (91.2) | 232 (96.7) |  |
| No | 136 (8.7) | 8 (3.3) |  |
| BMI (%) |  |  | 0.008 |
| <18.5 | 11 (0.5) | 1 (0.3) |  |
| 18.5-24.9 | 203 (13.1) | 26 (12.6) |  |
| 25-29.9 | 426 (31.2) | 48 (22.0) |  |
| ≥30.0 | 691 (55.3) | 136 (65.1) |  |
| Waist circumference * | 109.46 (0.75) | 112.23 (1.61) | <0.001 |
| Smoking (%) | 796 (55.6) | 153 (64.0) | 0.041 |
| Total cholesterol * | 4.87 (0.054) | 4.43 (0.097) | <0.001 |
| Triglycerides * | 2.20 (0.070) | 1.93 (0.095) | <0.001 |
| Uric acid * | 385.61 (4.14) | 397.92 (11.74) | <0.001 |
| Hypertension (%) | 1000 (67.1) | 203 (79.9) | <0.001 |
| Diabetes (%) | 410 (23.4) | 133 (52.9) | <0.001 |
| Cancer (%) | 277 (21.7) | 50 (25.8) | 0.851 |
| Stroke (%) | 123 (6.3) | 59 (24.5) | <0.001 |
| Coronary artery disease (%) | 124 (9.1) | 98 (44.8) | <0.001 |

*Data are presented as unweighted n (weighted percentage) for categorical variables and weighted means and standard errors for continuous variables.

**Table S4**. Odds ratios (95%CIs) for risk factors for concomitant HF in participants with gout.

| Variables | Adjust OR (95% CIs) | *P* value |
| --- | --- | --- |
| Age (years) | 1.03(1.00-1.05) | 0.036 |
| Female | 0.88 (0.58-1.33) | 0.529 |
| Mexican American (ref) |  |  |
| Other Hispanic | 3.04 (1.22-7.61) | 0.018 |
| Non-Hispanic White | 1.66(0.75-3.71) | 0.209 |
| Non-Hispanic Black | 3.15 (1.27-7.80) | 0.014 |
| Other race | 0.80 (0.20-3.19) | 0.749 |
| No insurance | 0.56 (0.22-1.39) | 0.205 |
| Less than 9th grade (ref) |  |  |
| 9-11th grade | 0.97 (0.45-2.12) | 0.948 |
| High school graduate | 0.90 (0.46-1.77) | 0.768 |
| College or AA degree | 0.59 (0.30-1.17) | 0.129 |
| College graduate or above | 0.29 (0.10-0.82) | 0.021 |
| Not a citizen of the US | 1.38(0.49-3.94) | 0.539 |
| Married/Living with partner (ref) |  |  |
| Widowed/divorced/separated | 1.80 (1.06-3.07) | 0.030 |
| Never married | 1.96 (0.99-3.90) | 0.054 |
| Hypertension | 0.97 (0.60-1.58) | 0.911 |
| Diabetes | 2.47 (1.67-3.65) | <0.001 |
| Cancer | 1.16 (0.69-1.95) | 0.564 |
| Stroke | 3.22 (1.87-5.53) | <0.001 |
| Coronary artery disease | 6.45 (3.94-10.53) | <0.001 |
| Obesity | 1.44 (0.86-2.40) | 0.159 |

CIs: confidence intervals; HF: heart failure.

**Table S5.** HR (95% CIs) for all-cause mortality according to heart failure and gout among participants in NHANES 2007-18.

| Variables | Adjusted HR (95% CIs) | *P* value |
| --- | --- | --- |
| No gout (ref) |  |  |
| Gout | 1.47 (1.31-1.65) | <0.001 |
| No gout or HF (ref) |  |  |
| Gout without HF | 1.34 (1.18-1.53) | <0.001 |
| HF without gout | 2.74 (2.44-3.09) | <0.001 |
| Gout with HF | 3.59 (2.93-4.38) | <0.001 |
| HF without gout (ref) |  |  |
| HF with gout | 1.45 (1.15-1.82) | 0.002 |
| HF without gout (ref) |  |  |
| HF and gout without HUA | 1.17 (0.83-1.65) | 0.36 |
| HF and gout with HUA | 1.78 (1.32-2.40) | <0.001 |

CIs: confidence intervals; HF: heart failure. HUA: hyperuricemia, HR: hazard ratios.

Age, gender, race and BMI were adjusted.

**Table S6**. HR (95% CIs) for cardiovascular mortality according to heart failure and gout among participants in NHANES 2007-18.

| Variables | Adjusted HR (95% CIs) | *P* value |
| --- | --- | --- |
| No gout (ref) |  |  |
| Gout | 1.53 (1.23-1.90) | <0.001 |
| No gout or HF (ref) |  |  |
| Gout without HF | 1.37 (1.05-1.79) | 0.019 |
| HF with gout | 4.56 (3.74-5.57) | <0.001 |
| Gout and HF | 5.11 (3.63-7.18) | <0.001 |

CIs: confidence intervals; HF: heart failure, HR: hazard ratios.
